# Supplementary material for: Climate change and intensive land use reduce soil animal biomass via dissimilar pathways
Source: eLife. 2020 Jul 28;9:e54749. doi: 10.7554/eLife.54749 (PMC7386910; doi:10.7554/eLife.54749)
Supplement: Supplementary file 1. — F-values are given and the significant effects are in bold font, with †= P < 0.1, * = P < 0.05, ** = P < 0.01, *** = P < 0.001. The predicted mean ± SD are given and different lowercase letters denote significant (P < 0.05) differences between land-use management intensities. [file elife-54749-supp1.doc]

**Supplementary File 1** Results from generalized linear mixed models with linear contrasts testing the effects of land-use management intensity within land-use types (‘croplands vs. grasslands’), croplands (‘conventional farming vs. organic farming’), grasslands (‘meadows vs. pastures’), and meadows (‘intensive meadows vs. extensive meadows’), respectively on (**A**) density of microarthropods, Acari, Oribatida (Orib_), Mesostigmata (Meso_), Prostigmata (Pros_), Entomobryidae (Ento_), Katiannidae (Kati_), Sminthuridae (Smin_), and (**B**) biomass of microarthropods, Acari, Oribatida (Orib_). F-values are given and the significant effects are in bold font, with † = *P* < 0.1, * = *P* < 0.05, ** = *P* < 0.01, *** = *P* < 0.001. The predicted mean ± SD are given and different lowercase letters denote significant (*P* < 0.05) differences between land-use management intensities.

| Fauna taxa | | Land-use type | | | |  | Croplands | | | |  | Grasslands | | | |  | Meadows | | | |
| --- | --- | --- | --- | --- | --- | --- | --- | --- | --- | --- | --- | --- | --- | --- | --- | --- | --- | --- | --- | --- |
|  |  | *F*-  value | Crop-  lands | vs | Grass  -lands |  | *F*-  value | Conventional | vs | Organic |  | *F*-  value | Meadows | vs | Pastures |  | *F*-  value | Intensive | vs | Extensive |
| (**A**) Density | Micro-  arthropods | **27.78**  ******* | 5030b  ±513 | ↑ | 8523a  ±419 |  | 0.82 | 4914a  ±726 | - | 5147a  ±726 |  | **4.97**  ***** | 9183a  ±513 | ↓ | 7202b  ±726 |  | 0.1 | 9019a  ±726 | - | 9348a  ±726 |
|  | Acari | **27.18**  ******* | 3748b  ±4589 | ↑ | 6833a  ±374 |  | 0.01 | 3697b  ±648 | - | 3799a  ±648 |  | **3.76**  ***** | 7355a  ±459 | ↓ | 5792a  ±648 |  | 0.12 | 7196a  ±648 | - | 7513a  ±648 |
|  | Orib_ | **18.45**  ******* | 2630b  ±380 | ↑ | 4739a  ±311 |  | 0.01 | 2598a  ±538 | - | 2661a  ±538 |  | **3.49**  ***** | 5149a  ±380 | ↓ | 3918a  ±538 |  | 0.26 | 5345a  ±538 | - | 4954a  ±538 |
|  | Meso_ | **20.31**  ******* | 954b  ±133 | ↑ | 1725a  ±108 |  | 0.00 | 962a  ±187 | - | 946a  ±187 |  | 1.65 | 1823a  ±133 | - | 1528a  ±187 |  | **5.32**  ***** | 1517b  ±187 | ↑ | 2128a  ±187 |
|  | Pros_ | **13.33**  ******* | 164b  ±44 | ↑ | 370a  ±36 |  | 0.42 | 136a  ±62 | - | 193a  ±62 |  | 0.24 | 382a  ±44 | - | 345a  ±62 |  | 1.22 | 334a  ±62 | - | 430a  ±62 |
|  | Ento_ | **10.55**  ****** | 62b  ±47 | ↑ | 255a  ±38 |  | 0.24 | 85a  ±67 | - | 40a  ±67 |  | 1.64 | 289a  ±48 | - | 187a  ±67 |  | 1.23 | 238a  ±67 | - | 340a  ±67 |
|  | Smin_ | **6.47**  ***** | 40b  ±46 | ↑ | 187a  ±38 |  | 0.02 | 45a  ±64 | - | 34a  ±64 |  | 2.35 | 226  ±46 | - | 108  ±64 |  | **7.04**  ***** | 345a  ±64 | ↓ | 108b  ±64 |
|  | Kati_ | **4.58**  ***** | 136a  ±40 | ↑ | 240a  ±33 |  | 0.00 | 136a  ±57 | - | 136a  ±57 |  | 0.48 | 255a  ±43 | - | 209a  ±58 |  | **5.81**  ***** | 345a  ±58 | ↓ | 164b  ±58 |
| (**B**) Biomass | Micro-  arthropods | **5.85**  ***** | 59.6b  ±7 | ↑ | 81.4a  ±5.7 |  | 0.08 | 57.6a  ±10 | - | 61.5a  ±10 |  | 2.48 | 87.7a  ±7 | - | 68.7a  ±10 |  | 0.39 | 83.4a  ±10 | - | 92.1a  ±10 |
|  | Acari | **3.13**  ***** | 48.8b  ±76.8 | ↑ | 64.2a  ±5.5 |  | 0.05 | 47.2a  ±9.5 | - | 50.3a  ±9.5 |  | 2.06 | 69.8a  ±6.7 | - | 53a  ±9.6 |  | 0.25 | 66.4a  ±9.6 | - | 73.2a  ±9.6 |
|  | Orib_ | **4.19**  ***** | 19.3b  ±5 | ↑ | 32.6a  ±4.1 |  | 0.1 | 17.7a  ±7.1 | - | 20.9a  ±7.1 |  | 2.3 | 37.1a  ±5 | - | 23.7a  ±7.3 |  | 0.08 | 35.7ba  ±7.1 | - | 38.6a  ±7.1 |
