# Supplementary material for: Climate change and intensive land use reduce soil animal biomass via dissimilar pathways
Source: eLife. 2020 Jul 28;9:e54749. doi: 10.7554/eLife.54749 (PMC7386910; doi:10.7554/eLife.54749)
Supplement: Supplementary file 2. — F-values are given and the significant effects are in bold font, with ** = P < 0.01, *** = P < 0.001. The predicted mean ± SD are given and different lowercase letters denote significant (P < 0.05) differences between the two seasons. [file elife-54749-supp2.docx]

**Supplementary File 2** Results from generalized linear mixed models with linear contrasts testing the effects of season (‘autumn vs. spring’) on (**A**) body size, (**B**) density, and (**C**) biomass of microarthropods, Acari, and Collembola. *F*-values are given and the significant effects are in bold font, with ** = *P* < 0.01, *** = *P* < 0.001. The predicted mean ± SD are given and different lowercase letters denote significant (*P* < 0.05) differences between the two seasons.

| Fauna taxa | (**A**) Body size | | | |  | (**B**) Density | | | |  | (**C**) Biomass | | | |
| --- | --- | --- | --- | --- | --- | --- | --- | --- | --- | --- | --- | --- | --- | --- |
|  | *F*-  value | Autumn | vs | Spring |  | *F*-  value | Autumn | vs | Spring |  | *F*-  value | Autumn | vs | Spring |
| Micro-  arthropods | 1.16 | 423a  ±11 | - | 442a  ±15 |  | **17.03**  ******* | 8194a  ±406 | ↓ | 4990b  ±623 |  | 0.45 | 75a  ±6 | - | 68a  ±8 |
| Acari | **20.05**  ******* | 342b  ±11 | ↑ | 415a  ±14 |  | **11.63**  ****** | 6366a  ±361 | ↓ | 4066b  ±543 |  | 0.34 | 56a  ±5 | - | 62a  ±8 |
| Collembola | 0.52 | 683a  ±26 | - | 652a  ±36 |  | **13.77**  ****** | 1828a  ±189 | ↓ | 924b  ±235 |  | **15.36**  ****** | 15a  ±1.4 | ↓ | 6.3b  ±1.9 |
