# Supplementary material for: Climate change and intensive land use reduce soil animal biomass via dissimilar pathways
Source: eLife. 2020 Jul 28;9:e54749. doi: 10.7554/eLife.54749 (PMC7386910; doi:10.7554/eLife.54749)
Supplement: Supplementary file 3. [file elife-54749-supp3.docx]

**Supplementary File 3** Descriptions of five land-use regimes.

| **Land-use regime**  (Abbreviation) | **Descriptions** |
| --- | --- |
| **Conventional farming**  (CF) | A typical regional crop rotation with the application of mineral fertilizers and pesticides. Crop rotational sequences: 2012-2013: oat (common crop on all subplots to homogenize soil conditions); 2013-2014: winter rape; 2014-2015: winter wheat; 2015-2016: winter barley. |
| **Organic farming**  (OF) | A crop rotation aiming to maintain soil fertility, minimize measures of pest and weed control, and provide an environmentally friendly management of agroecosystems with mechanical weed control, organic fertilization, non-stained seeds and restricted use of pesticides. Crop rotational sequences: 2012-2013: oat (common crop on all subplots to homogenize soil conditions); 2013-2014: horse bean; 2014-2015: winter wheat; 2015-2016: winter barley. |
| **Intensively-used meadow**  (IM) | Conventional used mixture of forage grasses with moderate fertilization and frequent mowing (3-4 times per year). |
| **Extensively-used meadow**  (EM) | A wide range of native common grasses, herbs and legumes (totally consisting 50 plant species, for each species seeds were sampled from different local populations to reflect to local gene pool and to introduce genetic variability) with moderate mowing (2-3 times per year) and no fertilization. |
| **Extensively-used pasture**  (EP) | Plant species composition and land management as the same as extensively used meadows (see above), but with sheep grazing (2-3 grazing periods per year with a group of 20 sheep grazing for 24 hours). |
